# Stacking order dynamics in the quasi-two-dimensional dichalcogenide 1*T*-TaS_2_ probed with MeV ultrafast electron diffraction

**DOI:** 10.1063/1.4982918

**Published:** 2017-05-03

**Authors:** L. Le Guyader, T. Chase, A. H. Reid, R. K. Li, D. Svetin, X. Shen, T. Vecchione, X. J. Wang, D. Mihailovic, H. A. Dürr

**Affiliations:** 1 SLAC National Accelerator Laboratory, 2575 Sand Hill Road, Menlo Park, California 94025, USA; 2 European XFEL GmbH, Holzkoppel 4, 22869 Schenefeld, Germany; 3 Department of Applied Physics, Stanford University, Stanford, California 94305, USA; 4 Jozef Stefan Institute and CENN Nanocenter, Jamova 39, SI-1000 Ljubljana, Slovenia

## Abstract

Transitions between different charge density wave (CDW) states in quasi-two-dimensional materials may be accompanied also by changes in the inter-layer stacking of the CDW. Using MeV ultrafast electron diffraction, the out-of-plane stacking order dynamics in the quasi-two-dimensional dichalcogenide 1*T*-TaS_2_ is investigated for the first time. From the intensity of the CDW satellites aligned around the commensurate *l* = 1/6 characteristic stacking order, it is found out that this phase disappears with a 0.3 ps time constant. Simultaneously, in the same experiment, the emergence of the incommensurate phase, with a slightly slower 2.0 ps time constant, is determined from the intensity of the CDW satellites aligned around the incommensurate *l* = 1/3 characteristic stacking order. These results might be of relevance in understanding the metallic character of the laser-induced metastable “hidden” state recently discovered in this compound.

## INTRODUCTION

Recent developments in the control of charge density waves (CDW), i.e., a combined periodic modulation of the electron density and a periodic lattice distortion, with either electrical current or femtosecond (fs) laser pulses could open the door for novel electronic devices (Yoshida *et al.*, 2015; Cho *et al.*, 2016; Ma *et al.*, 2016; Liu *et al.*, 2016; Vaskivskyi *et al.* 2014, 2016; and Stojchevska *et al.*, 2014). In particular, the 1*T* polytype of TaS_2_ displays a rich phase diagram, with the metallic normal (N) phase without CDW existing above T = 543 K, the incommensurate (I) phase with CDW order down to T = 354 K, the nearly commensurate (NC) phase down to T = 183 K and the insulating commensurate (C) phase below that (Ishiguro and Sato, 1991). Upon warming from the commensurate phase, a stripped discommensurate (T) phase is formed at 220 K and remains up to 280 K. In addition to these phases accessible via cycling the sample temperature, a novel metallic metastable “hidden” (H) phase was shown to form upon excitation by a single fs laser pulse (Vaskivskyi *et al.*, 2014, 2016 and Stojchevska *et al.*, 2014). While 1*T*-TaS_2_ is essentially thought of as 2-dimensional system, with a weak van der Waals interaction between adjacent layers, it is speculated that the insulating and conducting phase properties are driven by the layer stacking order in the 3rd dimension (Ritschel *et al.*, 2015). In the recent years, laser induced dynamics between different phases has been extensively studied with ultrafast electron diffraction (UED) focusing on transitions from the commensurate to nearly commensurate and from the nearly commensurate to incommensurate phases in 1*T*-TaS_2_ (Eichberger *et al.*, 2010; Han *et al.*, 2015; Haupt *et al.*, 2016; and Zhu *et al.*, 2015), 1*T*-TaSe_2_ (Sun *et al.*, 2015) and 4*H_b_*-TaSe_2_ (Erasmus *et al.*, 2012). However, all these studies were performed at normal incidence and therefore the stacking order dynamics has remained essentially unexplored.

Here, we investigate how and on which time scale the stacking order evolves upon femtosecond laser excitation. Using the 3.3 MeV electron diffraction setup at SLAC (Weathersby *et al.*, 2015), we probe the ultrafast dynamics of the commensurate to incommensurate phase transition upon excitation by a 50 fs, 800 nm wavelength laser pulse. Taking advantage of the nearly flat Ewald sphere for 3.3 MeV electrons, we measured simultaneously the different dynamic behavior of the set of CDW satellite reflections at various wave vectors along the *l*-Bragg rod.

## EXPERIMENTAL METHODS

**FIG. 1.**
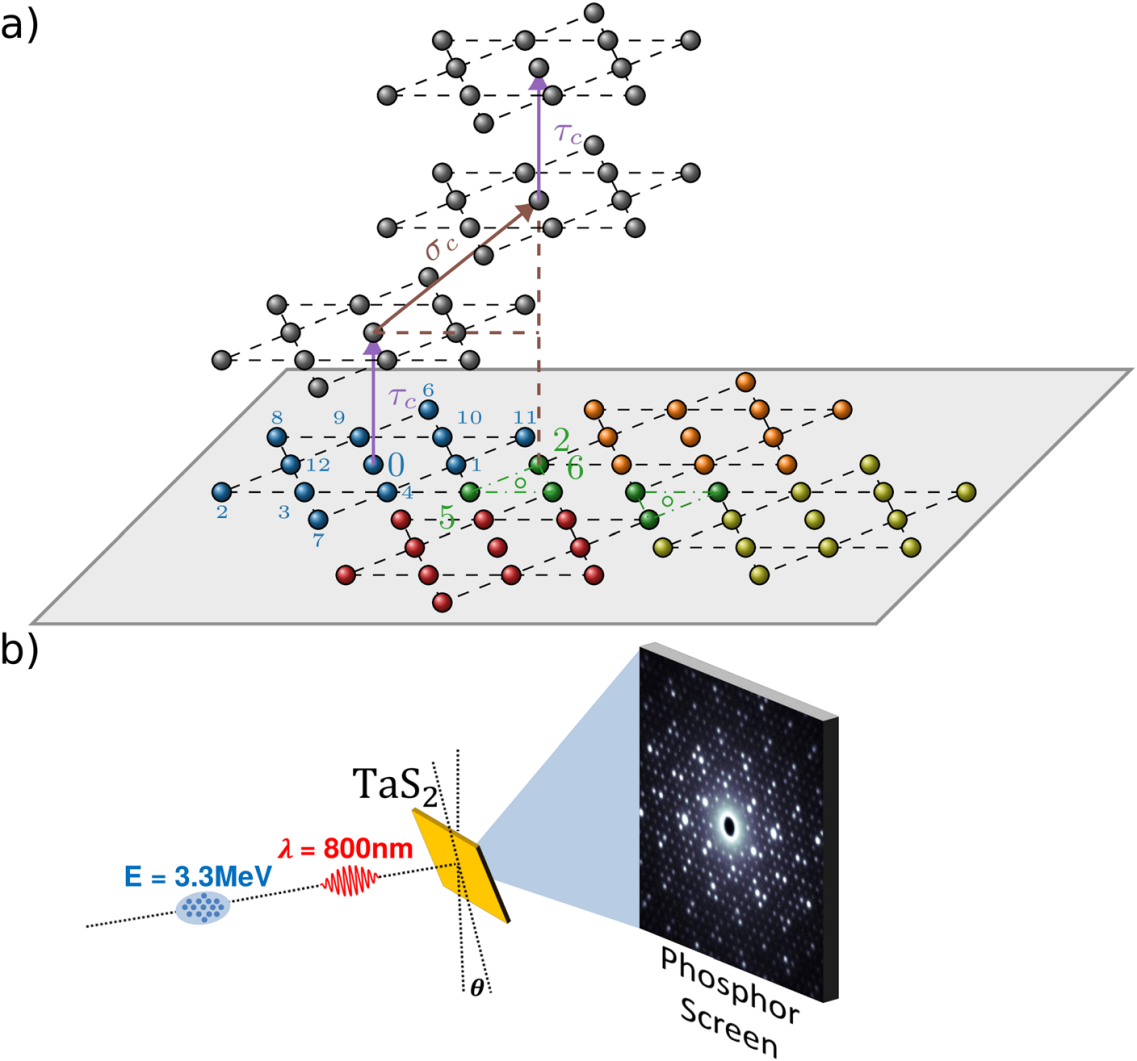
(a) Direct space view of the star of David formation in the basal plane, where the 13 Ta atoms are numbered in sequence. The out-of-plane layer stacking for the commensurate phase is a succession of a τ_c_ translation, positioning the star of David on top of each other in position 0, followed by a random selection between 3 possible σ_c_ translations positioning the star of David on top of positions 2, 5, or 6. In the nearly commensurate or incommensurate phase, the ⟨σ_h2_⟩ or ⟨σ_I_⟩ stacking, respectively, is a three times repeat of a translation linking the center of a star of David and the center of the 2-5-6 green triangle. After three such translations, the layer is back in the 0 position. (b) Schematic of the time-resolved electron diffraction experiment, where the sample is tilted by an angle θ from normal incidence to probe the out-of-plane layer stacking changes between the commensurate and nearly commensurate or incommensurate phase. The 800 nm wavelength 50 fs laser pulse excites the TaS_2_ sample. After a delay t, the 3.3 MeV 100 fs electron bunch diffracts from the sample on a phosphor screen.

Time-resolved electron diffraction measurements were conducted at the UED setup at SLAC, whose detailed description is given elsewhere (Weathersby *et al.*, 2015). Essentially, 3.3 MeV electron bunches are sent at a 180 Hz repetition rate through a thin 1*T*-TaS_2_ sample. The diffracted electron beam is detected at a 3 m travel distance after the sample on a phosphor screen, which is imaged with an Andor camera recording the diffraction pattern, as depicted in Fig. 1. The 800 nm wavelength pump laser employed to excite the sample is focused down to a spot size of 1 mm FWHM (Full Width at Half Maximum). At the same time, the electron probe spot size is kept at least 3 times smaller at 300 *μ*m FWHM. The sample temperature can be varied between 35 K and 360 K. The sample can be rotated from its normal by angles up to θ = ±10° without blocking the electron beam by the sample mount, allowing us to investigate the out-of-plane ordering dynamics. Electron diffraction patterns can be recorded at variable pump-probe delay with acquisition times of a few seconds.

The free-standing sample investigated here was prepared by first exfoliating flakes from a TaS_2_ single crystal sample in a Gel-Pack**^®^** box. These flakes were then transferred on a poly(methyl methacrylate) (PMMA) film on glass. Atomic force microscopy (AFM) was used to determine the thickness of these flakes, and the one used in this study was found to be 60 nm thick. The PMMA with TaS_2_ flakes was then dissolved in acetone, and the floating flakes were caught on a transmission electron microscope (TEM) copper grid and finally washed in isopropyl alcohol (IPA).

## RESULTS AND DISCUSSION

**FIG. 2.**
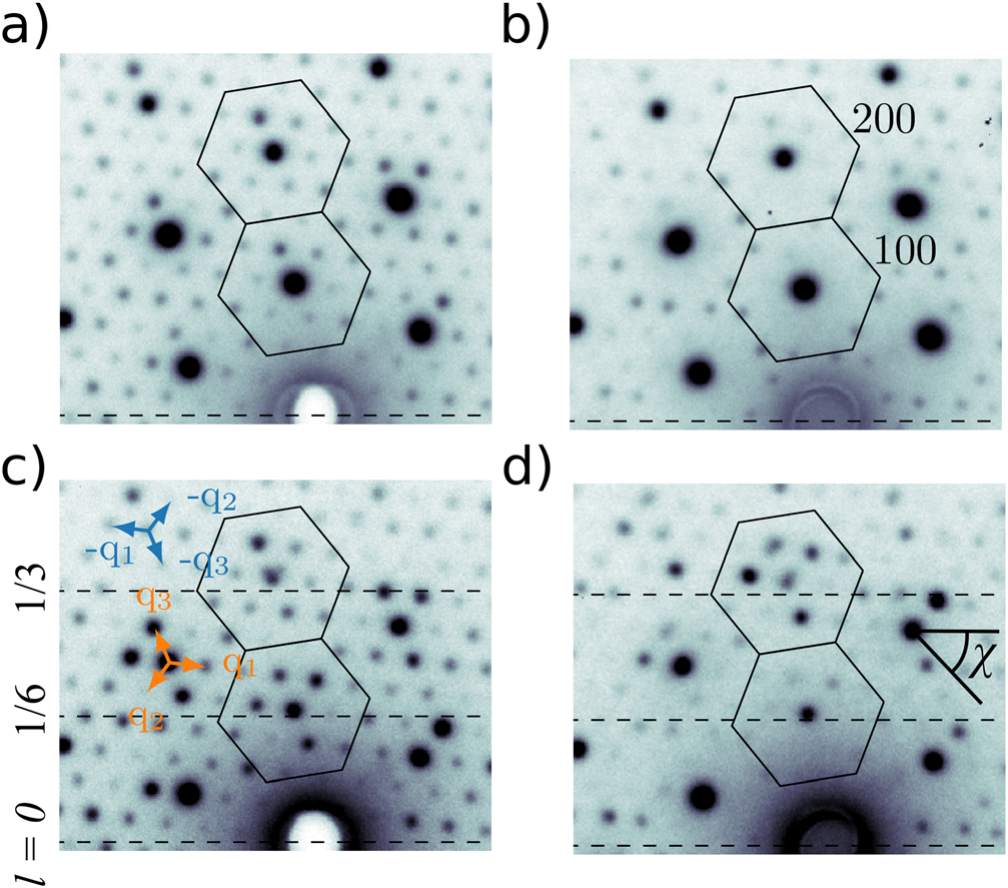
Part of the electron diffraction pattern recorded at normal incidence (a) in the commensurate phase at T = 150 K and (b) in the nearly commensurate phase at T = 300 K, as well as for the case of a tilt angle of θ = 5° from normal incidence at (c) T = 150 K and (d) T = 300 K. The first Brillouin zones around the 100 and 200 Bragg peaks are shown as the hexagons. The 3 positive first order satellites q_1_, q_2_, and q_3_ are shown as orange arrows, while the 3 negative satellites of the CDW −q_1_, −q_2_, and −q_3_ are shown as blue arrows. For the tilted case, dashed lines at *l* = 0, 1/6, and 1/3 are shown. In (d), the azimuth angle χ is shown. In all cases, the data are shown on a logarithm scale.

An electron diffraction pattern from our 1*T*-TaS_2_ sample is shown in Fig. 2(a) for a sample tilt θ = 0° from normal incidence and a temperature T = 150 K in the commensurate phase. The first Brillouin zone around the **100** and **200** Bragg peaks are shown as hexagons surrounding them. The 6 first order satellites q_1_ and −q_1_, q_2_ and −q_2_, q_3_ and −q_3_ of the triple CDW in 1*T*-TaS_2_ are visible around each central Bragg peak and correspond to the star-of-David formation, where 13 Ta atoms cluster together, as shown in Fig. 1(a). Upon increasing the temperature to 300 K in the nearly commensurate phase, the intensity of all the first order satellites vanishes, as shown in Fig. 2(b). To understand this behavior, one should first consider the changes occurring in the basal plane. In the commensurate phase, the CDW satellites form an angle φ = 13.9° in the basal plane with the *hk0* reciprocal lattice. This angle changes abruptly at the commensurate to nearly commensurate phase transition by about −2°, after which it slowly reduces with temperature in the nearly commensurate phase until the nearly commensurate to incommensurate phase transition is reached where it changes abruptly to φ = 0° (Ishiguro and Sato, 1991). However, this rotation of the CDW satellites in the basal plane cannot explain the vanishing of the satellites intensity observed at normal incidence. To understand this, it is necessary to consider the change in out-of-plane stacking order occurring between the commensurate phase and the nearly commensurate phase by tilting the sample. An electron diffraction pattern recorded for a sample tilt of θ = 5° from normal incidence is shown in Fig. 2(d) for the nearly commensurate phase at T = 300 K. Here, three bright first order satellites, namely −q_1_, −q_2_ and −q_3_, appear around the **200** Bragg peak, which for θ = 5° is near *l* = 1/3. At the same time, the first order satellite remain very weak around **100** Bragg peak, which for θ = 5° is near *l* = 1/6. Upon cooling to 150 K in the commensurate phase, this changes as the 3 first order satellites −q_1_, −q_2_ and −q_3_ are now visible around **100**, while the ones around **200** become weaker. This behavior suggests a change of stacking order from an *l* = 1/3 to an *l* = 1/6.

**FIG. 3.**
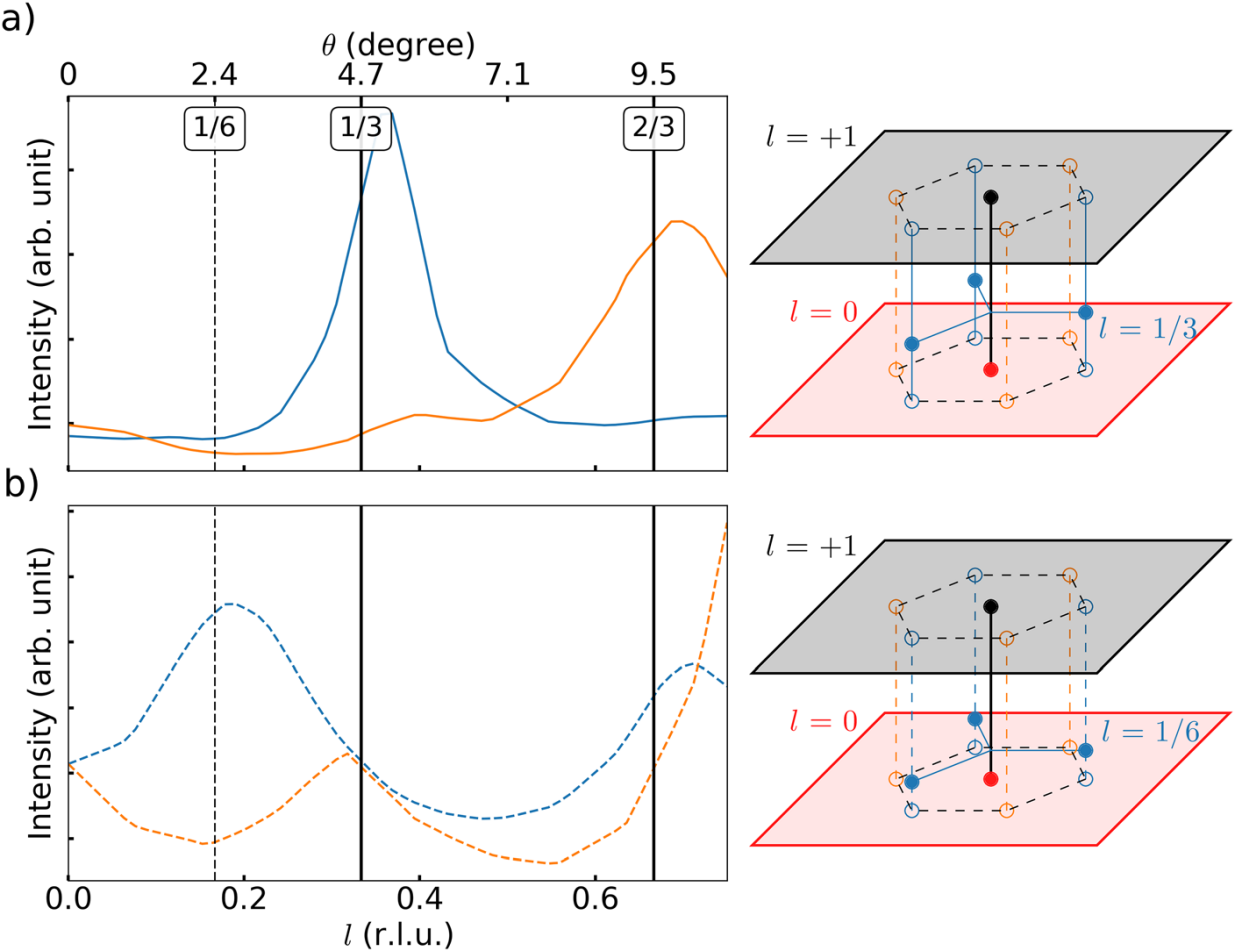
Averaged intensity of the 3 positive CDW satellites in orange and 3 negative satellites in blue as function of the Miller index *l* and corresponding incidence angle θ for the **200** Bragg peak, for (a) the nearly commensurate phase at T = 300 K and for (b) the commensurate phase at T = 150 K, together with a schematic in each case of the CDW in reciprocal space with the filled blue satellites at either *l* = 1/3 or l = 1/6 for the nearly commensurate or commensurate phase, respectively. The open orange and blue satellite circles are the projection on the *l* = 0 and 1 plane which are seen at normal incidence.

To determine further the equilibrium out-of-plane stacking order in our sample, the tilt was varied and the averaged intensity of the three positive (q_1_, q_2_, q_3_) and three negative (−q_1_, −q_2_, −q_3_) CDW satellites around **200** are shown in Fig. 3(a) as dashed orange and blue lines, respectively, as a function of the Miller index *l* for the nearly commensurate phase at T = 300 K. Here, we clearly see a peak around *l* = 1/3 for the negative CDW satellites and the symmetric behavior with a peak at *l* = 2/3 = −1/3 for the positive CDW satellites. Following Nakanishi and Shiba (1984) naming convention, this is in accordance with the ⟨σ_h2_⟩ stacking found for the nearly commensurate phase which is a 3 times repeat of the same in-plane translation, as depicted in Fig. 1(a) with the green open circle, connecting the center of a star-of-David formation and the center of the 2-5-6 green triangle (Nakanishi and Shiba, 1984). This arrangement corresponds in the reciprocal space to satellite intensity narrowly located around *l* = 1/3. Upon cooling to the commensurate state as shown in Fig. 3(b) for T = 150 K, the peaks significantly broaden and shift to *l* = 1/6 for the negative satellites and to *l* = 5/6 = −1/6 for the positive satellites with an additional smaller peak at *l* = 1/3 and *l* = 2/3 = −1/3, respectively. This is compatible with a change from the ordered ⟨σ_h2_⟩ stacking to the partly disordered (τ_c_, σ_c_) staking, which is a succession of an in-phase stacking τ_c_ followed by a random selection between 3 different translated stacking σ_c_, as depicted in Fig. 1(a) (Tanda *et al.*, 1984 and Nakanishi and Shiba, 1984). This demonstrates that for this particular tilt angle θ = 5°, we are both sensitive to the commensurate stacking order with *l* = 1/6 around the **100** Bragg peak and to the nearly commensurate stacking order with *l* = 1/3 around **200** Bragg peak simultaneously. Next, we will address how this change in out-of-plane stacking order dynamically occurs when the system changes from the commensurate to the nearly commensurate phase.

**FIG. 4.**
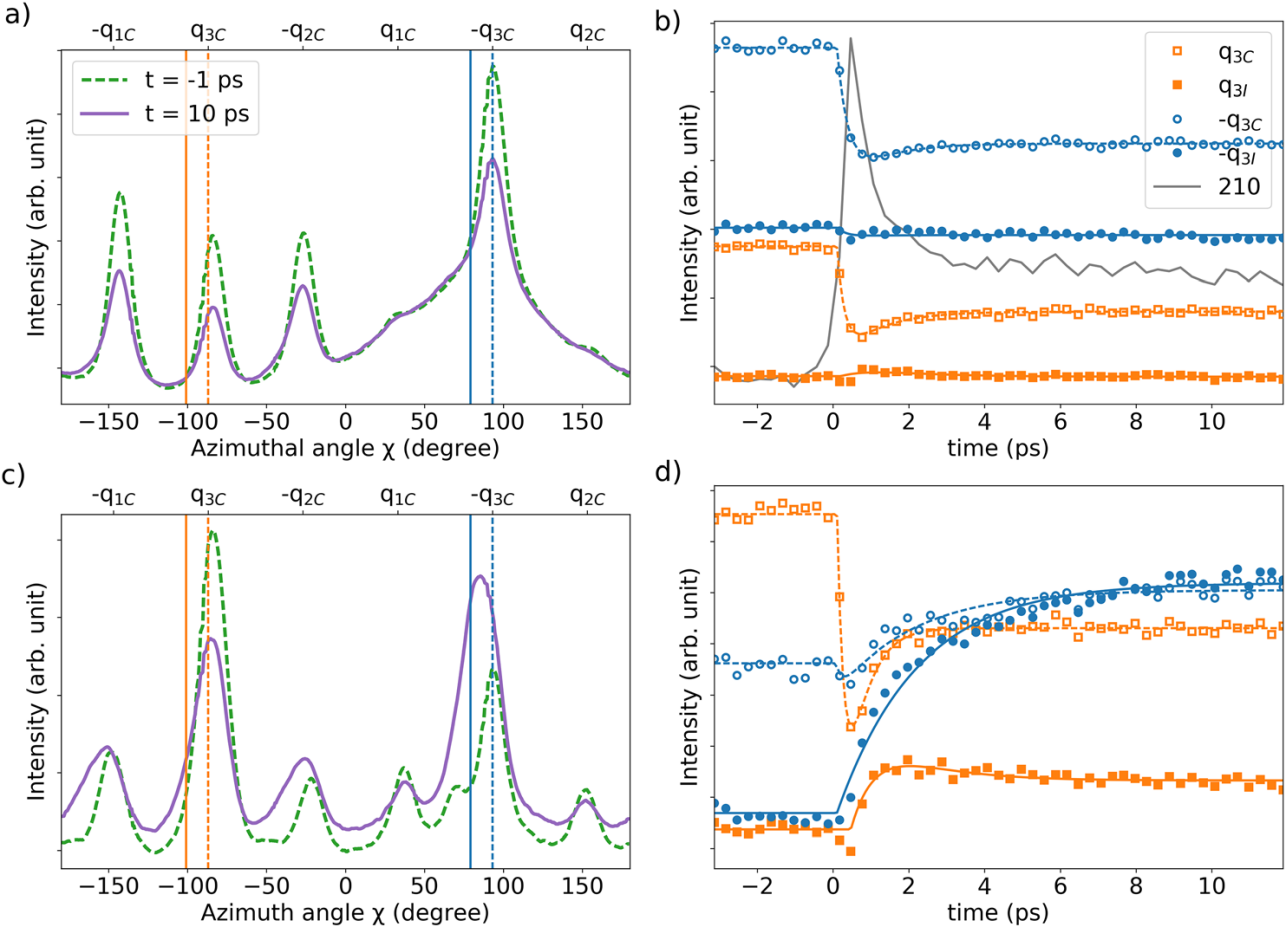
(a) Intensity profile of the 6 first order CDW satellites around the **100** Bragg peak for a negative delay at t = −1 ps in dashed green line and a positive delay at t = 10 ps in a continuous purple line. The vertical dashed blue (orange) line indicates the position in the commensurate phase of the −q3 (q_3_) satellite −q_3C_ (q_3C_), while the continuous blue (orange) line indicates the position for the incommensurate phase of −q_3_ (q3) satellite −q_3I_ (q_3I_). (b) Time evolution after laser excitation of the CDW satellites intensity at the commensurate (open symbols, dashed lines) and incommensurate position (filled symbols, continuous lines) for the q_3_ (orange) and −q_3_ (blue) satellites, where the lines correspond to a fit of the data points with a two exponential time constants. The grey data point are the **210** Bragg peak intensity at *l* = 0. (c) and (d) are for the **200** Bragg peak. In all figures, the sample temperature is T = 140 K, the laser fluence is F = 3.0 mJ/cm^2^ and the incidence angle is θ = 5°.

To investigate how this staking order dynamically changes, time-resolved pump-probe measurements were performed near the commensurate to nearly commensurate phase transition at a sample base temperature of 140 K with a laser pump fluence of F = 3.0 mJ/cm^2^ and an incidence angle of *θ* = 5°. The diffracted intensity distribution around the **100** Bragg peak as function of the azimuth angle χ, as defined in Fig. 2(d), at negative delay t = −1 ps and positive delay t = 10 ps, are shown in Fig. 4(a). It can be seen here that all the 6 satellites intensity decrease upon excitation. For the q_3_ (−q_3_) satellites, the time dependent intensity around the commensurate position q_3C_ (−q_3C_) with φ = 13.9° and incommensurate position q_3I_ (−q_3I_) with φ = 0° are shown in Fig. 4(b) as open and closed symbols, respectively. The intensity at the commensurate position decreases for both q_3_ and −q_3_ with a 0.3 ps characteristic time constant and remains constant afterwards up to 12 ps, as extracted from a two exponential fit function

$$A\left(1-B\left(1-e^{-(t-t_{0})/\tau_{B}}\right)+C\left(1-e^{-(t-t_{0})/\tau_{C}}\right)\right),$$

where $\tau_{B}(\tau_{C})$ is bound to be smaller (larger) than 0.5 ps, respectively. At the same time, no dynamics are observed at the incommensurate position q_3I_ and −q_3I_. However, the time dependence of the **210** Bragg intensity at *l* = 0 shown in Fig. 4(b) displays an increase in the same time scale. Indeed, as the diffracted intensity is conserved, if the CDW is weakened, the Bragg intensity should increase, as previously observed (Eichberger *et al.*, 2010; Haupt, 2016; and Erasmus, 2012). From this information alone, one would be tempted to conclude that the laser pulse is weakening the CDW, resulting in a decrease of the diffracted intensity of the corresponding satellites without inducing any particular phase transition.

However, the picture changes drastically when looking at the **200** Bragg peak, for which the satellites profiles before and after the laser pulse excitation are shown in Fig. 4(c). It is evident there that the −q_3_ satellite is both more intense and rotated towards the incommensurate position after laser excitation. This increase of intensity, as opposed to the decrease observed around the **100** Bragg peak, is unambiguously indicative of phase transition, here a change in stacking order from the commensurate phase at *l* = 1/6 to either the nearly commensurate or incommensurate phase at *l* = 1/3, the position around which this **200** Bragg peak is aligned. The time dependence of −q_3I_ incommensurate satellite, as shown in Fig. 4(d), is characterized by a strong increase with a characteristic time constant of $\tau_{C}=$ 2.0 ps, as extracted from a fit with two exponential time constants, while the amplitude of exponential with the shorter time constant $\tau_{B}$ is negligible. At the same time, the −q_3C_ commensurate satellite shows a moderate increase which could be due to the increase seen at the −q_3I_ incommensurate satellite and the limited q-resolution in the recorded UED patterns. The latter broadens the satellites to a peak width that is of the order of the commensurate satellite rotation φ = 13.9° and originates from the electron bunch emittance, sample flatness and crystal quality. The q_3C_ commensurate satellite, which is aligned on the *l* = 1/3 side peak of the partly disordered (τ_c_, σ_c_) stacking of the commensurate phase, as shown in Fig. 3(b), shows a significant decrease and is therefore another indication that the commensurate phase is disappearing. The q_3I_ incommensurate satellite shows a moderate intensity increase with a faster time constant than for the −q_3I_ incommensurate satellite which seems to be related with the overall increase in diffuse scattering of the excited sample seen as an increased background in Fig. 4(c).

## CONCLUSION

In summary, using MeV electron diffraction, the stacking order dynamic in 1*T*-TaS_2_ was for the first time investigated. Thanks to the flat Ewald sphere displayed by MeV electrons, several CDW satellites could be investigated simultaneously, characterizing both the initial commensurate phase and the excited incommensurate phase. We evidenced that the commensurate phase disappears with a characteristic time constant of 0.3 ps, while the incommensurate phase emerges with a slower 2.0 ps time constant. It is crucial to realize that all these data are recorded simultaneously and not in essentially different experiments, as it would be the case with hard X-rays diffraction for example. Moreover, with an improved q-resolution, it would become possible to discern the commensurate and the nearly commensurate satellite intensity as well as the “hidden” phase, opening the path to the study of their in-plane and out-of-plane ordering dynamic and metallic character.
